# Consumption of Vitamin A Rich Foods and Their Primary Dietary Sources among Rural Preschoolers in the Gambella Region, Southwest Ethiopia

**DOI:** 10.4314/ejhs.v35i4.2

**Published:** 2025-07

**Authors:** Dessalegn Tamiru, Gorden Koang Dak, Beyene Wondafrash

**Affiliations:** 1 Department of Nutrition and Dietetics, Faculty of Public Health, Jimma University, Ethiopia

**Keywords:** Vitamin A, Vitamin A-Rich Foods, Preschool Children, Nutritional Intake, Ethiopia

## Abstract

**Background:**

Globally, the consumption of vitamin A-rich foods remains a significant public health challenge, particularly in sub-Saharan Africa, where access to these foods is limited. Preschool-aged children in rural areas are especially vulnerable to vitamin A deficiency due to poor dietary diversity and limited access to healthcare services. In Ethiopia, there is a scarcity of research on vitamin A intake among rural preschoolers, particularly in the Gambella Region. The objective of this study was to assess the consumption patterns and primary dietary sources of vitamin A-rich foods among rural preschool children in the Gambella Region of Ethiopia.

**Methods:**

A community-based cross-sectional study was conducted among 381 preschool children in the Gambella Region. Data were collected using the Helen Keller International (HKI) food frequency questionnaire and a 24-hour dietary recall. Statistical analysis was performed using SPSS version 26.0, with findings presented through frequency tables and graphs to illustrate dietary trends.

**Results:**

Of the 381 participants, 380 (99.7%) completed the study. The majority of children (74.3%) ate two meals per day, predominantly cereals and legumes. Only 32.4% consumed vitamin A-rich foods, with particularly low intake reported for milk (2.9%), dark green leafy vegetables (6.1%), fish (5%), and fortified margarine (0.5%). Over 75% of children had inadequate vitamin A intake (P < 0.01), and 78.4% of caregivers lacked awareness of vitamin A-rich food sources. Nearly half of households (48.1%) relied on personal production for these foods, and 54.9% indicated that availability was seasonal.

**Conclusion:**

The study highlights critically low consumption of vitamin A-rich foods among rural preschool children in the Gambella Region, placing them at increased risk of vitamin A deficiency and related health complications such as impaired vision and weakened immunity. To address this issue, targeted nutrition education and community awareness programs are essential, alongside policy interventions to improve food access and dietary diversity.

## Introduction

Over the past few decades, global efforts have significantly reduced the prevalence of inadequate micronutrient intakes, including vitamin A deficiency (1). However, sub-Saharan Africa remains an exception, largely due to widespread dependence on micronutrient-poor staple grains in the food supply (2). In low- and middle-income countries, approximately 28% of children do not achieve the minimum dietary diversity required to meet their vitamin A needs, especially during the complementary feeding period (3). In Ethiopia, consumption of vitamin A-rich foods remains low; only 14% of children under the age of three consume fruits, vegetables, or animal-source foods rich in vitamin A. Moreover, just 26% of children aged 6-35 months meet the recommended intake of vitamin A-rich foods (4). Recent data indicate that the availability of vitamin A in the national food supply is insufficient, contributing to widespread inadequate intake among infants and young children (4, 5).

In developing countries, vitamin A intake often falls short, particularly among vulnerable groups such as young children, pregnant women, and individuals from socioeconomically disadvantaged backgrounds (5, 6, 7). Preschool children in low-income settings are particularly at risk due to their higher nutritional demands. Globally, about one-third of preschool-aged children suffer from subclinical vitamin A deficiency (VAD), with Africa and Southeast Asia bearing the highest burden. Approximately 0.9% of children experience night blindness, and a 2013 review estimated that 29% of children aged six months to five years are vitamin A deficient (3, 5, 6, 7, 8, 9).

Vitamin A deficiency is a major public health concern in many low-income countries, contributing to increased child mortality rates (10). VAD affects over half of all countries worldwide, with the most severe impact in Africa and Southeast Asia, particularly among preschool children and pregnant women. In Africa, more than 30 million children under the age of five are affected. Globally, an estimated 190 million children do not meet the recommended vitamin A intake. According to WHO, 250 million preschool children suffer from VAD each year, and between 250,000 to 500,000 of them go blind annually (3, 10, 14, 15, 18, 19, 20-23). In Africa, 44.4% of preschool children are at risk, accounting for over one-third of the global burden of childhood xerophthalmia. In Ethiopia, vitamin A deficiency causes approximately 80,000 deaths annually and affects 61% of preschool-aged children, with a 28% prevalence recorded in 2017—the second highest globally. Poor dietary intake of vitamin A-rich foods remains the leading cause of VAD among preschool children (3, 10, 14, 15, 18, 19, 20–23).

Eliminating VAD is essential to reducing child mortality, potentially by as much as 50%. A diet rich in carotene and retinol—such as dark green leafy vegetables and animal-source foods—is both effective and cost-efficient (9, 10, 11, 12). The most recent Demographic and Health Survey (DHS) in East Africa reported poor vitamin A consumption among preschool children, with Ethiopia recording a 64.14% rate of inadequate intake compared to 19.56% in Burundi (21, 22). Despite ongoing efforts to reduce VAD, it continues to pose a significant public health threat in Ethiopia, where fewer than 10% of children consume sufficient amounts of vitamin A-rich fruits and vegetables (22). While many African countries, including Ethiopia, emphasize vitamin A supplementation programs, there is often a lack of initiatives promoting sustainable dietary intake. Moreover, most studies in Ethiopia have focused on dietary diversity among women or vitamin A intake in children under two, leaving older preschool-aged children largely overlooked. No prior research has examined the patterns of vitamin A consumption and food accessibility among rural preschool children in the Gambella Region of Ethiopia.

## Methods and Materials

**Study area and setting**: This study was conducted in the Itang Special District (ISD) of the Gambella Region, western Ethiopia, from March to September 2023. Itang is situated 45 km west of Gambella town and is bordered by the Oromia Region to the northeast, Abobo Woreda to the south, Gambella Zuria to the east, and Lara to the west (23). The district has a total population of 50,156, with 24,576 (48.99%) being male (24). Of the 23 kebeles in the district, 22 are rural and one is urban. About 95% of the population resides in the floodplain of the Baro River basin, with the remaining 5% leading a semi-nomadic lifestyle. Most villages are located near the Baro River, which plays a vital role in the area's ecology and the residents' livelihoods (23, 24, 25). The district experiences a tropical climate, with annual temperatures ranging from 24°C to 41°C, and covers an area of 2,188 km^2^. Rivers like the Baro contribute significantly to the region's biodiversity and water supply (26).

**Study population**: The study targeted the 8,344 rural households within the Itang Special District. The study population included mothers or caregivers of preschool children aged 2–6 years residing in rural kebeles.

**Inclusion and exclusion criteria**: Children aged 2–6 years living in rural households within selected kebeles of Itang District, and who had a mother or caregiver present during the survey, were included. Children who had not resided in the area for at least six months were excluded.

**Sample size determination**: The required sample size was calculated using a single population proportion formula, assuming a 62% prevalence of insufficient vitamin A-rich food consumption among Ethiopian children (27), a 95% confidence level, and a 10% non-response rate. The initial sample size of 398 was adjusted to 381 using a correction formula due to the target population being under 10,000.

**Sampling procedure**: A multi-stage sampling approach was employed. Itang Special District was purposively selected from the 14 districts in the Gambella Region due to its remoteness. Seven kebeles were randomly selected, and households were chosen using systematic sampling. A sampling interval of 21 was calculated by dividing the total source population (8,344) by the final sample size (381). The sample was proportionally allocated across the selected kebeles. In households with more than one eligible child, one was selected randomly using a lottery method. If no eligible respondent was found, the next household was visited. Household lists were obtained from local health posts.

**Data collection procedures**: Data were collected using the Helen Keller International (HKI) Food Frequency Questionnaire and a 24-hour dietary recall. The HKI FFQ assesses the frequency of consumption of vitamin A-rich foods over the past week and is culturally adapted to local diets (28). Caregivers were asked to recall all foods and beverages given to their preschool children in the previous 24 hours. The 24-hour dietary recall method is known for its ability to provide detailed, quantitative dietary information (29).

The HKI method calculates a weighted vitamin A consumption score by summing the number of days vitamin A-rich foods from animal and plant sources were consumed. Adequate intake is defined as consuming animal-source vitamin A foods on more than four days per week, or a combined total of animal and plant sources on more than six days per week (28, 29).

Data collectors received three days of training at Itang Health Center. A pre-test was conducted in Itang town to validate the tools, and necessary adjustments were made. The questionnaire was translated into the local language and then back-translated into English to ensure consistency.

**Data processing and analysis**: Data were checked for completeness and consistency before being entered into EpiData and exported to SPSS version 26.0 for analysis. Descriptive statistics, including frequencies, percentages, and means, were used to summarize socio-demographic characteristics and dietary intake. Q–Q plots were used to assess data normality. Summary tables and graphs were used to present the results. Descriptive statistics also measured the adequacy of vitamin A-rich food consumption, offering insights into related influencing factors.

**Ethical considerations**: Ethical clearance was obtained from the Research Ethics Committee of Jimma University, Institute of Health, School of Public Health (IHRPG1/1249/22). Approval letters were also secured from the Itang Special District Administration and the district executive office. Informed verbal and written consent was obtained from all participants after explaining the study's objectives, procedures, and potential benefits. Participants were informed that their participation was voluntary and that they could withdraw from the study at any time without penalty.

## Results

Out of 381 selected participants, 380 (99.7%) completed the study. More than half of the respondents (55.3%) lived in households with 1–4 members. The majority (80%) of respondents were the biological mothers of the preschool children, and almost all households (96.6%) were male-headed. Most caregivers (84.5%) were female, and a significant proportion (58.9%) of mothers were not employed (Table 1).

**Table 1:** Socio-demographic characteristics of study participants, 2023

Variables	Categories	Frequency (N =380)	Percent
**Household size**	1-4	210	55.3
	5-9	70	18.4
	>9	100	26.3
**Ethnic group**	Nuer	345	90.8
	Anuak	5	1.32
	Others	30	7.89
**Household Head**	Male	367	96.6
	Female	13	3.4
**Sex of child**	Male	136	35.8
	Female	244	64.2
**Child age in year**	2-3	84	22.1
	4-5	177	46.6
	6	119	31.3
**Gender of caregiver**	Male	59	15.5
	Female	321	84.5
**Caregiver relation to child**	Mother	304	80.0
	Father	14	3.7
	sibling	14	3.7
	Grandparents	31	8.2
	Others	17	4.5
**Marital status**	Marriage	319	83.9
	Single	8	2.1
	Window	32	8.4
	Divorce/Separated	21	5.5
**Caregivers education**	Not read & write	238	62.6
	Primary school	88	23.2
	Secondary school	24	6.3
	Diploma & above	30	7.9
**Religion of caregiver**	Protestant	299	78.7
	Catholic	9	2.4
	Orthodox	16	4.2
	Muslim	4	1.1.
	Adventists	52	14.6
**Maternal Occupation**	Not employed	224	58.9
	Government employee	65	17.1
	Self-employee	91	23.9

Meal frequency was generally low, with 74.3% of preschool children consuming only two meals per day. Cereals and legumes were the dominant food groups, particularly at breakfast (66.7%) and lunch (42.3%). Vitamin A-rich fruits and vegetables were rarely consumed, with only 5.5% of children eating them at breakfast and 16% at lunch. At dinner, cereals remained the predominant food group, consumed by 38.6% of children, while vitamin A-rich foods were consumed far less frequently (Table 2).

**Table 2:** Meal consumption pattern of preschool children, 2023

Variable	Categories	Frequency	Percent
Meal frequency per day	2 times	283	74.3
	3 times	89	23.4
	4 times	8	2.1
Breakfast consumption regularly	Yes	69	18.1
	No	311	81.6
Breakfast composition	Cereals	254	66.7
	Fish and Meat products	66	17.3
	Dark Green leafy vegetables	29	7.6
	Fruits and vegetables	21	5.5
	Milk and milk products	10	2.6
Lunch composition	Cereals	161	42.3
	Fish and Meat products	63	16.5
	Dark Green leafy vegetables	29	7.6
	Fruits and vegetables	61	16.0
	Milk or milk product	66	17.3
Dinner composition	Cereals	147	38.6
	Fish and Meat products	79	20.7
	Dark Green leafy vegetables	43	11.3
	Fruits and vegetables	56	14.7
	Milk and milk product	55	14.4

Overall, just 32.4% of preschool children consumed vitamin A-rich foods. Only 2.89% of children consumed dairy products such as cheese and butter, and 6.05% consumed dark green leafy vegetables. Fish, including species rich in vitamin A such as king mackerel and salmon, were consumed by only 5%. Other vitamin A-rich foods were also minimally consumed: 2.63% ate sweet potatoes, 1.32% consumed pumpkins, 1.84% ate mangoes, and only 3.16% consumed eggs or chicken. The least consumed were vitamin A-fortified foods like margarine, consumed by just 0.53% of children (Table 3).

**Table 3:** Vitamins a food groups consumed by preschool children, 2023

Type of vitamin A-rich foods	Frequency N=380	Percent (%)
Dark Green leafy vegetables (DGRV)	23	6.05
Sweet Potatoes	10	2.63
Pumpkin	5	1.32
Mangoes	7	1.84
Watermelon	8	2.11
Carrots	9	2.37
Eggs	7	1.84
Chicken	7	1.84
Cow goat or sheep liver	5	1.32
Fish	19	5
Milk and milk products	11	2.89
Kale	10	2.63
Vitamin A-fortified food like Margarine	2	0.53
Total vitamin A-rich foods consumption	123	32.4

**Figure 1:**
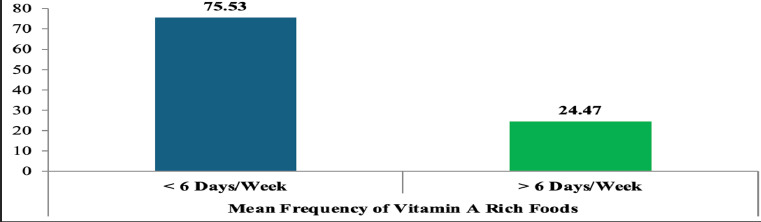
Mean frequency consumption of Vitamin A-rich foods, 2023 (P<0.01)

Using the Helen Keller International (HKI) 7-day food frequency questionnaire, the mean frequency of vitamin A-rich food consumption was calculated. More than three-quarters (75.53%) of preschool children had a total consumption frequency of fewer than six days per week, indicating inadequate intake according to HKI and WHO standards (Figure 1).

In terms of awareness, 67.9% of caregivers had never heard of vitamin A, and 78.4% had no knowledge of foods rich in vitamin A. When asked to name vitamin A-rich foods, only 25.6% could identify examples such as mangoes, pumpkins, carrots, sweet potatoes, and lemons. A small proportion (25.8%) reported providing such foods to their children, primarily for eye health (Table 4).

**Table 4:** Knowledge/Awareness of caregivers about vitamin A rich foods, 2024

Variable	Categories	Frequency (N=380)	Percent
**Ever heard about vitamin A**	Yes	123	32.1
	No	258	67.9
**Had knowledge of vitamin A-rich foods**	Yes	82	21.6
	No	298	78.4
**Sources of Vitamin A-rich foods**	Fish Liver and organ meats	51	62.2
	Mangoes, pumpkins, carrots etc	21	25.6
	Guava Pepper	10	12.2
**Know benefits of Vitamin A rich food**	Yes	60	15.8
	No	320	84.2
**Benefit of Vitamin A Rich Foods**	For health child	26	1
	Only food available/afford to buy	13	3.5
	For a vision of Child	16	4.3
	Don't know	320	85.3

Regarding sources, 45.8% of respondents obtained vitamin A-rich plant-based foods from their own production, while 31.3% relied on local markets. More than half (54.9%) reported that these foods—particularly fish and fruits—were seasonally available, most often between February and June (Table 5).

**Table 5:** Sources and availability of Vitamin A rich food in Itang Special District, Gambella region, 2024

Variables	Category of respond	Frequency (N=380)	Percent
Animal Sources of Vitamin - A Rich Foods	Buy fish, milk or eggs from the market	172	45.4
	Keep chicken for Eggs or Meat	86	22.6
	Keep Cows, Sheep/goats for milk or meat	97	25.5
	Buy Meat/liver from Butchery	18	4.74
	Food Aid	7	1.8
Plant Sources of Vitamin-A-Rich Foods	Grow Vegetables in my farm	174	45.8
	Buy from the local or nearer market	119	31.3
	Grow fruits in my farm or home garden	39	10.3
	Buy them from a distance market	48	12.6
Season of vitamin A rich foods availability	February -June	209	54.9
	July-October	149	39.4
	November -Jan	22	5.77

## Discussion

This study assessed the consumption patterns and dietary sources of vitamin A-rich foods among rural preschool children in Itang Special District, Gambella Region, Southwest Ethiopia. The findings revealed alarmingly low intake levels during a critical stage of development. Inadequate vitamin A intake during early childhood, pregnancy, and lactation can lead to serious health consequences, including vitamin A deficiency syndrome (30–35).

Only 32.4% of preschool children in this study consumed vitamin A-rich foods, a figure lower than reported in Addis Ababa (36%) (32), Ghana (85%) (33), the Ethiopian DHS report (39%) (27), and systematic reviews of children aged 6–23 months (38.99%) (22). These differences may be attributed to variations in children's age groups, geographical settings, parental knowledge and awareness, and socioeconomic status.

The low intake identified in this study emphasizes the urgent need for targeted interventions in rural areas. Poor education, limited media access, low income, and prevailing food taboos limit both the knowledge and availability of vitamin A-rich foods. Many households live in small, thatched-roof structures that cannot accommodate media equipment, limiting access to health education. These findings are supported by existing literature that highlights how poor food production, limited market access, large family sizes, and low maternal awareness further worsen the risk of vitamin A deficiency (36).

The consumption rate of 32.4% represents a significant public health concern, particularly given vitamin A's role in supporting growth, immune function, and vision. The limited intake of animal-source foods, green leafy vegetables, and fortified products suggests that underlying structural issues—such as poverty, inadequate food supply chains, and low dietary diversity—are influencing these outcomes. Sociocultural beliefs, seasonal food shortages, and lack of nutrition knowledge compound these challenges. Addressing these factors will require multi-faceted interventions that focus on nutrition education, improving agricultural productivity, and enhancing access to diverse food sources (23, 25–30).

Furthermore, 75.53% of children consumed vitamin A-rich foods fewer than six days per week, placing them below HKI and WHO/FAO minimum thresholds (37). This finding is slightly better than a study in Sodo Zuria, Southern Ethiopia, where 98% had similarly low intake (38), but worse than studies in Uganda (80%) (39), Kachabira District (28.8%) (40). Differences may stem from ecological factors—Gambella's tropical environment supports fruit and fish production, while urban slums or drought-prone areas may not. Seasonal variation in food availability also plays a role, with fruits and fish more accessible from January to June, and vegetables mainly available during the July-November rainy season. Any intervention must consider these seasonal patterns, encouraging off-season cultivation or preservation methods to maintain year-round access to vitamin A-rich foods (35–39).

Meal frequency was also low, with 74.3% of children eating only twice a day. Their diets were dominated by cereals and legumes, which, while providing essential macronutrients, are insufficient for meeting micronutrient needs. The limited inclusion of vitamin A-rich foods like fruits, vegetables, and animal products contributes to hidden hunger and nutrient deficiencies. Interventions should aim to diversify children's diets by promoting local production and raising awareness among caregivers about balanced nutrition (34–40).

A major strength of this study is its focus on an understudied age group—children aged 2 to 6 years. Most prior studies in Ethiopia have centered on children under two or on pregnant and lactating women. Additionally, the use of the HKI food frequency method, validated for assessing both intake and risk of vitamin A deficiency, enhances the reliability of the findings. However, the study had some limitations. A mixed-methods approach could have provided deeper insights into caregivers' cultural beliefs about food. Moreover, recall bias may have affected the accuracy of dietary data, as caregivers sometimes struggled to recall food items consumed over the previous week or 24-hour period.

In conclusion, this study found that only 32.4% of rural preschool children in Itang Special District consumed vitamin A-rich foods, with 75.53% having an average intake of fewer than six days per week. There was a significant knowledge gap, as 67.9% of caregivers had never heard of vitamin A and 78.4% were unaware of foods rich in the nutrient. While 25.6% could identify some vitamin A-rich foods like mangoes and carrots, only 25.8% reported giving such foods to their children, mostly due to perceived eye health benefits. Nearly half (45.8%) of households sourced these foods from their own production, but seasonal availability and limited market access (31.3%) hindered consistent intake. Overall, vitamin A consumption in the district is well below HKI and WHO/FAO thresholds, placing children at high risk for vitamin A deficiency. Immediate, multi-sectoral interventions—including community education, food system improvements, and targeted nutrition programs—are essential to reduce the burden of VAD in this vulnerable population.
